# The Inhibition Effect of the Seaweed Polyphenol, 7-Phloro-Eckol from *Ecklonia Cava* on Alcohol-Induced Oxidative Stress in HepG2/CYP2E1 Cells

**DOI:** 10.3390/md19030158

**Published:** 2021-03-17

**Authors:** Liyuan Lin, Shengtao Yang, Zhenbang Xiao, Pengzhi Hong, Shengli Sun, Chunxia Zhou, Zhong-Ji Qian

**Affiliations:** 1 School of Chemistry and Environment, Shenzhen Institute of Guangdong Ocean University, College of Food Science and Technology, Guangdong Ocean University, Zhanjiang 524-088, China; liyuanlin1024@163.com (L.L.); 15766385620@163.com (S.Y.); xzhenbang@163.com (Z.X.); hongpengzhigdou@163.com (P.H.); xinglsun@126.com (S.S.); chunxia.zhou@163.com (C.Z.); 2 Southern Marine Science and Engineering Guangdong Laboratory, Zhanjiang 524-088, China

**Keywords:** 7-phloro-eckol, HepG2/CYP2E1 cells, oxidative stress, apoptosis

## Abstract

The liver is vulnerable to oxidative stress-induced damage, which leads to many diseases, including alcoholic liver disease (ALD). Liver disease endanger people’s health, and the incidence of ALD is increasing; therefore, prevention is very important. 7-phloro-eckol (7PE) is a seaweed polyphenol, which was isolated from *Ecklonia cava* in a previous study. In this study, the antioxidative stress effect of 7PE on HepG2/CYP2E1 cells was evaluated by alcohol-induced cytotoxicity, DNA damage, and expression of related inflammation and apoptosis proteins. The results showed that 7PE caused alcohol-induced cytotoxicity to abate, reduced the amount of reactive oxygen species (ROS) and nitric oxide (NO), and effectively inhibited DNA damage in HepG2/CYP2E1 cells. Additionally, the expression levels of glutathione (GSH), superoxide dismutase (SOD), B cell lymphoma 2 (Bcl-2), and Akt increased, while γ-glutamyltransferase (GGT), Bcl-2 related x (Bax), cleaved caspase-3, cleaved caspase-9, nuclear factor-κB (NF-κB), and JNK decreased. Finally, molecular docking proved that 7PE could bind to BCL-2 and GSH protein. These results indicate that 7PE can alleviate the alcohol-induced oxidative stress injury of HepG2 cells and that 7PE may have a potential application prospect in the future development of antioxidants.

## 1. Introduction

The liver is the main organ of alcohol metabolism, and the adverse reaction of alcohol metabolism will damage the liver [1]. The main cause of alcoholic liver disease (ALD) is long-term excessive drinking. ALD symptoms, including alcohol fatty liver disease and alcohol hepatitis, can further lead to steatohepatitis, liver fibrosis, cirrhosis, and the most severe form of liver cancer [2]. In China, liver disease affects about 300 million people, and the number of cases of ALD is increasing, with a major impact on the global burden of liver disease [3]. Fat accumulation in the liver occurs in the early stages of ALD, and only this stage can be reversed without any medical intervention; therefore, early diagnosis and proper treatment of ALD are essential before irreversible liver damage occurs [4].

Due to the production of reactive oxygen species (ROS) during alcohol metabolism, the liver is vulnerable to oxidative stress-induced injury [5]. Oxidative stress induced by free radicals has been reported to play a key role in the degeneration, inflammation, apoptosis, and necrosis of hepatocytes [6]. ROS molecules are highly active and play an important role in cell functions but are also closely related to pathology. High levels of ROS can cause cell death by damaging the cell structure by oxidation of nucleic acids, proteins, and lipids [7]. Nitric Oxide (NO) is also involved in a wide range of toxic oxidative reactions with ROS [8]. Therefore, inhibiting the level of reactive oxygen species may be a way to prevent ALD. Among them, ROS can be eliminated by antioxidant metalloenzymes, such as superoxide dismutase (SOD) [9]. Glutathione (GSH), as an important antioxidant, can scavenge free radicals in the body [10]. The activity of γ -glutamyltransferases (GGT) also can be used as a marker for ALD evaluation [11,12]. Moreover, when free radicals damage the kidney, the inflammatory reaction, which is usually the mechanism of protection and repair, will appear and may stimulate the formation of other free radicals [13]. In addition, ROS can act as the second messengers of intracellular signal transduction cascades and regulate the expression of apoptotic genes through MAPK activation, thus increasing apoptosis. It is reported that Bcl-2 related x (Bax) proteins related to caspase-3 and B cell lymphoma 2 (BCL-2) play a key role in apoptosis [14]. Apoptosis can also be activated by other signal molecules [8], such as Akt [15], nuclear factor-κB (NF-κB) [16], and JNK [17], a member of mitogen-activated protein kinase.

In recent decades, a large number of highly effective and low-toxicity marine active substances have been discovered in the vast ocean [18]. Among them, seaweed, as one of the important plants in the marine, has a variety of active components and has been widely studied [19,20]. The active ingredients of seaweed include Sulfated seaweed polysaccharides [21], polyphenols [22], proteins [23], terpenes [24], alkaloids [25], phenolic compounds [26], and halogenated compounds [27]. Among these ingredients, seaweed polyphenols are considered a good source of antioxidants [28]. 7-phloro-eckol (7PE), a seaweed polyphenol, was extracted from edible brown algae, *Ecklonia cava* [29], and its structure was similar to eckol and dieckol. It is noteworthy that eckol and dieckol have been reported to have anticancer [30,31] and antioxidant [31,32,33,34,35] properties and modulate anti-monoamine oxidases [36], but the role of 7PE has received little attention. Therefore, 7PE, with a structure similar to eckol and dieckol, has high research value.

In order to prove that 7PE can be used as a potential preventive substance against oxidative stress, ethanol-induced oxidative stress in HepG2/CYP2E1 cells was used as a mature model [37,38]. Our data suggest that 7PE inhibits ethanol-induced oxidative stress, which indicates that 7PE has antioxidant potential and is expected to be the source of antioxidant development in the future.

## 2. Results

### 2.1. Effects of 7PE on Cell Viability of HepG2/CYP2E1 Cells

**Figure 1:**
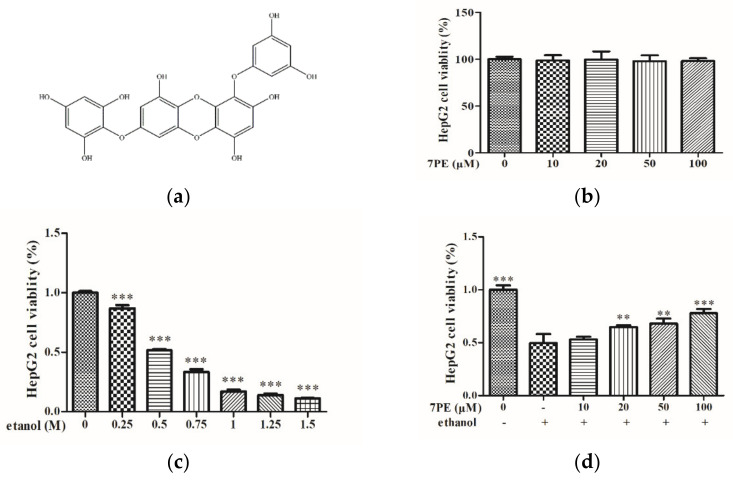
Effect of 7-phloro-eckol (7PE) on cell viability of HepG2/CYP2E1 cells. (**a**) Chemical structure of 7PE from marine brown alga, *Ecklonia cava*. Effect of 7PE (0, 10, 20, 50, and 100 µM) on the viability of HepG2/CYP2E1 cells; (**b**) HepG2/CYP2E1 cells were evaluated by MTT assay, respectively; (**c**) the viability of various doses of ethanol (0, 0.25, 0.5, 0.75, 1.0, 1.25, and 1.5 M) on HepG2/CYP2E1 cells; (**d**) protective effects of 7PE (0, 10, 20, 50, and 100 µM) on ethanol-induced (0.5 M) HepG2/CYP2E1 cell injury. Data are shown as mean ± SD (*n* = 3). * Compared with the control group (ethanol-induced group). ** *p* < 0.01; *** *p* < 0.001.

The results showed no significant change in the viability of HepG2/CYP2E1 cells (Figure 1b), which indicated that there was no toxic effect of 7PE treatment of up to 100 μM. Thus, the employed concentrations (0, 10, 20, 50, and 100 μM) of 7PE were used in all the subsequent experiments. Figure 1c shows that ethanol decreased cell viability in a dose-dependent manner. Cell viability was approximately 50% when cells were exposed to 0.5 M ethanol. As depicted in Figure 1d, treatment with 7PE significantly increased the viability of HepG2/CYP2E1 cells following exposure to 0.5 M ethanol. The results showed that 7PE (20, 50, and 100 μM) could effectively prevent damage to HepG2/CYP2E1 cells from ethanol.

### 2.2. Determination of Intracellular ROS and NO

**Figure 2:**
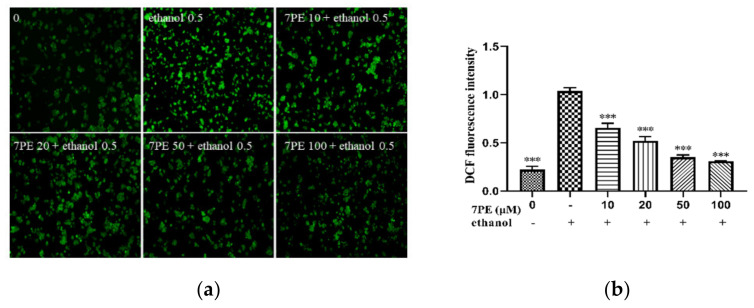

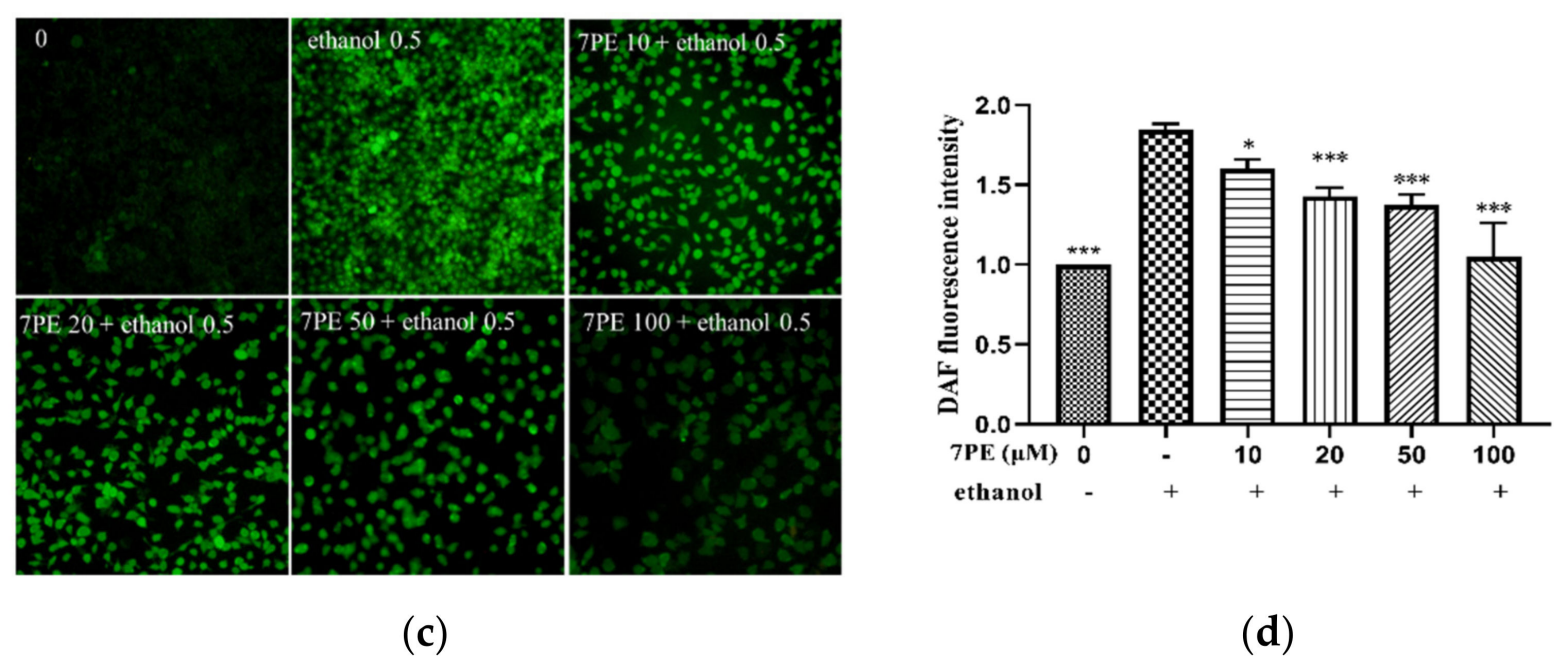
Effect of 7PE on intercellular reactive oxygen species (ROS) and (NO) generation in HepG2/CYP2E1 cells damaged by ethanol. (**a**) Then, the cells were exposed to 2,7-dichlorodi-hydrofluorescein diacetate (DCFH-DA) for 30 min. DCF fluorescence of the treated cells was measured using an inverted fluorescence microscope; (**b**) the relative DCF fluorescence intensity analysis of image; (**c**) the cells were exposed to 3-amino,4-aminomethyl-2′,7′-difluorescein diacetate (DAF-FM-DA) for 30 min. DAF fluorescence of the treated cells was measured using an inverted fluorescence microscope; (**d**) the relative DAF fluorescence intensity analysis of the images. Data are shown as mean ± SD (*n* = 3). * Compared with the control group (ethanol-induced group). * *p* < 0.05; ** *p* < 0.01; *** *p* < 0.001.

The cells were treated as shown in Figure 2, then treated with 2,7-dichlorodi-hydrofluorescein diacetate (DCFH-DA) and 3-amino,4-aminomethyl-2′,7′-difluorescein diacetate (DAF-FM-DA), respectively, for 30 min, and an inverted fluoroscope was used to obtain Figure 2a,c. In the blank group, there was no significant fluorescence. On the other hand, in the control group, high ROS levels were observed. Treatment with different concentrations of 7PE for 2 h downregulated ROS levels in a dose-dependent manner. The result of NO is similar (Figure 2c,d). These results show that 7PE had a protective effect against alcohol-induced cytotoxicity in HepG2/CYP2E1 cells by inhibiting ROS and NO.

### 2.3. Determination of Intracellular DNA Damage

**Figure 3:**
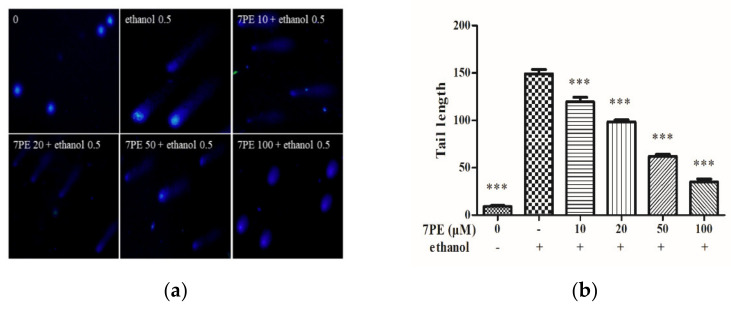
(**a**) The cells were stained with DAPI. Images were obtained using an inverted fluorescence microscope; (**b**) tail lengths of the comets were analyzed by CASP. HepG2/CYP2E1 cells without treatment formed the blank group. Data are shown as mean ± SD (*n* = 3). * Compared with the control group (ethanol-induced group). ** *p* < 0.01; *** *p* < 0.001.

Cells were obtained by comet assay with DAPI and then imaged using an inverted fluorescence microscope to obtain Figure 3. In the blank group, there was no obvious tailing fluorescence. In the control group, HepG2/CYP2E1 cells showed obvious tailing fluorescence in 0.5 M ethanol. However, with the increase in 7PE concentration, the length of the comet tail decreased, which proves that 7PE could prevent alcohol-induced oxidative damage at the cellular level.

### 2.4. Effect of 7PE on the Level of Oxidative Stress-Related Proteins

**Figure 4:**
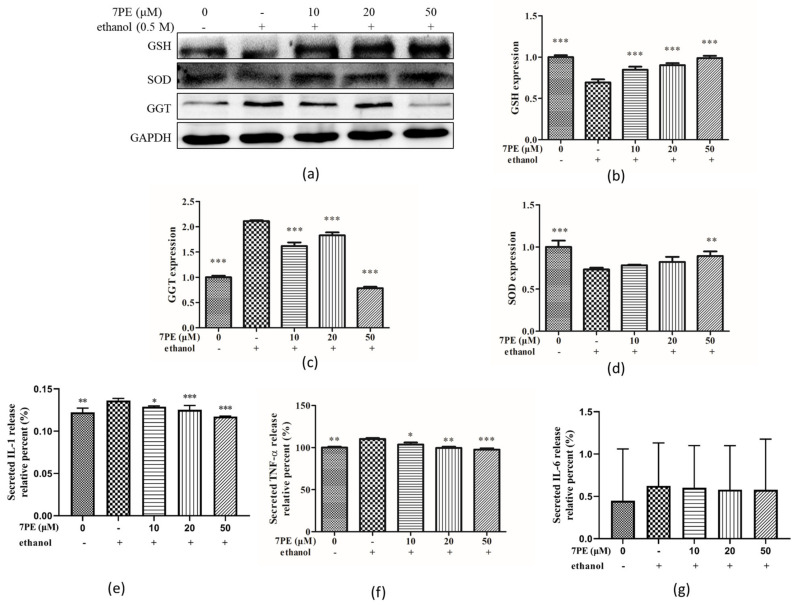
Effect of 7PE on superoxide dismutase (SOD), glutathione (GSH), and γ-glutamyl transferase (GGT) protein levels by Western blot and detection of related inflammatory factors by ELISA in HepG2 cells. Cells were treated with 7PE (10, 20, and 50 μM) for 2 h and then treated with 0.5 M ethanol for 24 h. GAPDH was used as an internal control. (**a**) Protein expression (relative to GAPDH) was evaluated; (**b**) GSH protein expression was evaluated; (**c**) GGT protein expression was evaluated; (**d**) SOD protein expression was evaluated; (**e**) interleukin-1 (IL-1) expression was evaluated; (**f**) tumor necrosis factor-α (TNF-α) expression was evaluated; (**g**) IL-6 expression was evaluated. Data are shown as mean ± SD (*n* = 3). * Compared with the control group (ethanol-induced group). * *p* < 0.05; ** *p* < 0.01; *** *p* < 0.001.

As shown in Figure 4a–d, the protein levels of GSH and SOD in the control group decreased significantly, and the protein levels of the GGT increased significantly in the control group. In addition, compared with the control group, after treatment with 7PE, the protein levels of GSH and SOD increased significantly and were dose-dependent, while the protein level of GGT decreased.

The results of ELISA showed that the levels of interleukin-1 (IL-1), IL-6, and tumor necrosis factor-α (TNF-α) in the control group were higher than those in the blank group (Figure 4e–g). Then, compared with the control group, the inflammatory factors IL-1 and TNF-α decreased after 7PE treatment, but IL-6 did not change significantly.

### 2.5. Detection of Related Apoptosis Proteins

**Figure 5:**
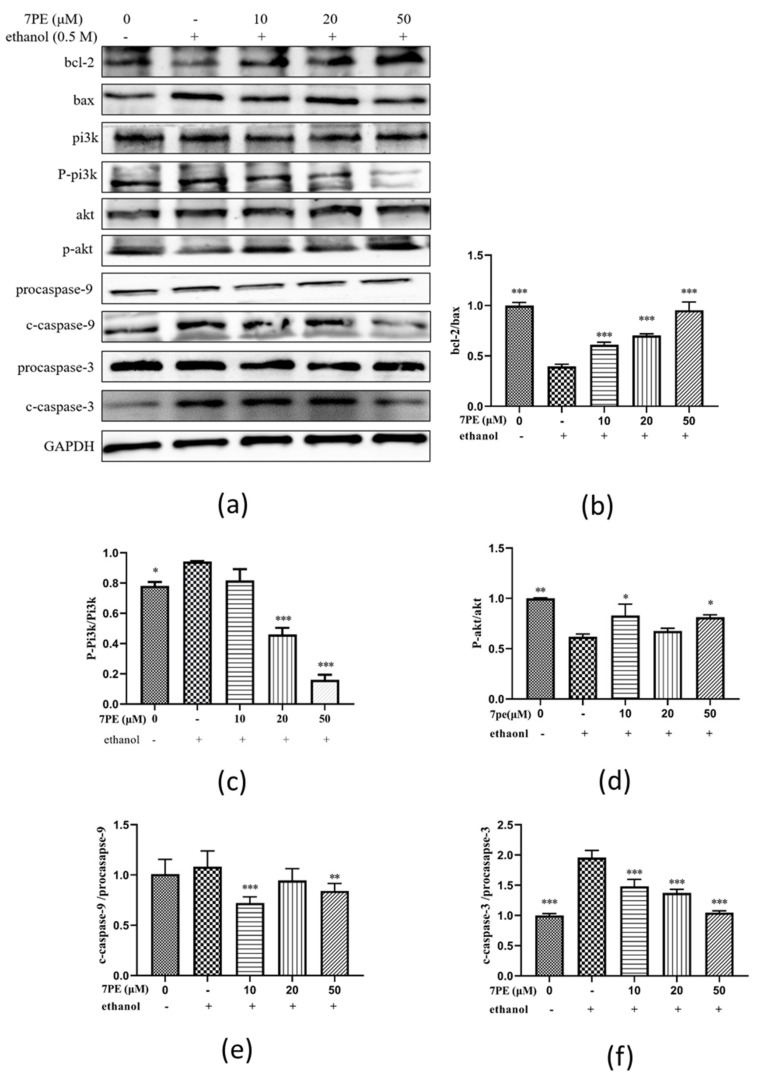
The effect of 7PE on the levels of related apoptosis proteins in HepG2/CYP2E1 cells treated with ethanol (0.5 M) by Western blot. GAPDH was used as an internal control. (**a**) Western blot analysis of bcl-2, bax, pi3k, p-pi3k, akt, p-akt, caspase-9, cleaved caspase-9, caspase-3 (c-3), and cleaved caspase-3 (c-c-3) protein levels; (**b**) the ratios of bcl-2 and bax proteins were calculated; (**c**) the ratios of p-pi3k and pi3k were calculated; (**d**) the ratios of p-akt and akt were calculated; (**e**) the ratios of cleaved caspase-9 and procaspase-9 were calculated; (**f**) the rations of cleaved caspase-3 and procaspase-3 were calculated. Data are shown as mean ± SD (*n* = 3). * Compared with the control group (ethanol-induced group). * *p* < 0.05; ** *p* < 0.01; *** *p <* 0.001.

In order to determine whether 7PE has an anti-apoptotic effect on alcohol-induced cytotoxicity in HepG2/CYP2E1 cells, the expressions of Bcl-2 and Bax were determined (Figure 5). In comparison with the blank group, the expression of the bcl-2 protein decreased, and the expression of bax protein increased in the control group. Compared with the control group, after 7PE treatment, the expression of bcl-2 increased while the expression of bax decreased in a dose-dependent manner. Additionally, compared with the blank group, the p-pi3k protein, cleaved caspase-9 (c-c-9) protein, and cleaved caspase-3 (c-c-3) protein in the control group increased, while p-akt decreased (Figure 5c–f). These results indicate that 7PE could alleviate the oxidative stress induced by ethanol by regulating the production of apoptosis-related proteins.

### 2.6. Effect of 7PE on the NF-κB Signal Pathway

**Figure 6:**
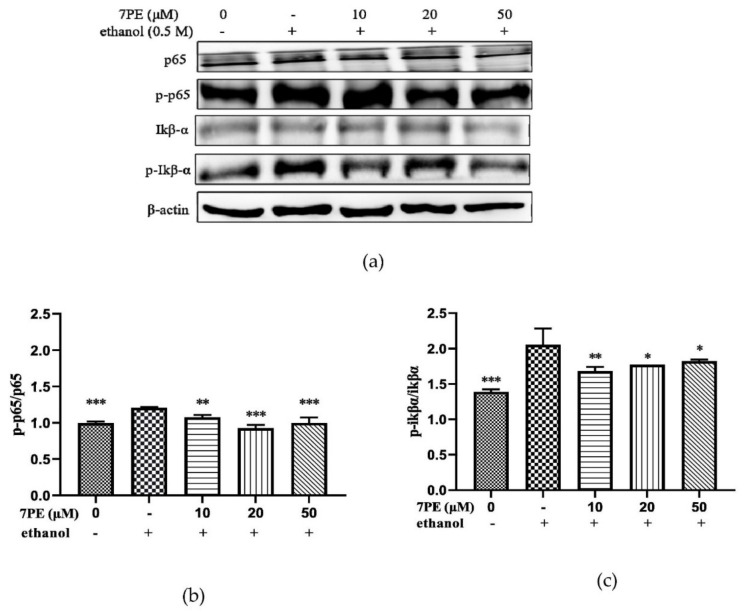
(**a**) The phosphorylation levels of p65, p-p65, IκBα, and p-IκBα proteins in HepG2/CYP2E1 cells. Cells were treated with 7PE (10, 20, and 50 μM) for 2 h, then treated with 0.5 M ethanol for 24 h; (**b**) the ratios of p-p65/p65 were calculated; (**c**) the ratios of p-IκBα/IκBα were calculated. Data are shown as mean ± SD (*n* = 3). * Compared with the control group (ethanol-induced group). * *p* < 0.05; ** *p* < 0.01; *** *p* < 0.001.

The effect of 7PE on the NF-κB signal pathway was studied. As shown in Figure 6, compared with the blank group, the phosphorylation levels of p65 and IκBα in the control group were significantly increased. After 7PE treatment, the values of p-p65/p65 and p-IκBα/IκBα showed a dose-dependent decrease (Figure 6b,c). This indicated that 7PE inhibited the phosphorylation of NF-κB at the protein level to inhibit apoptosis.

### 2.7. JNK and p53 Protein Levels

**Figure 7:**
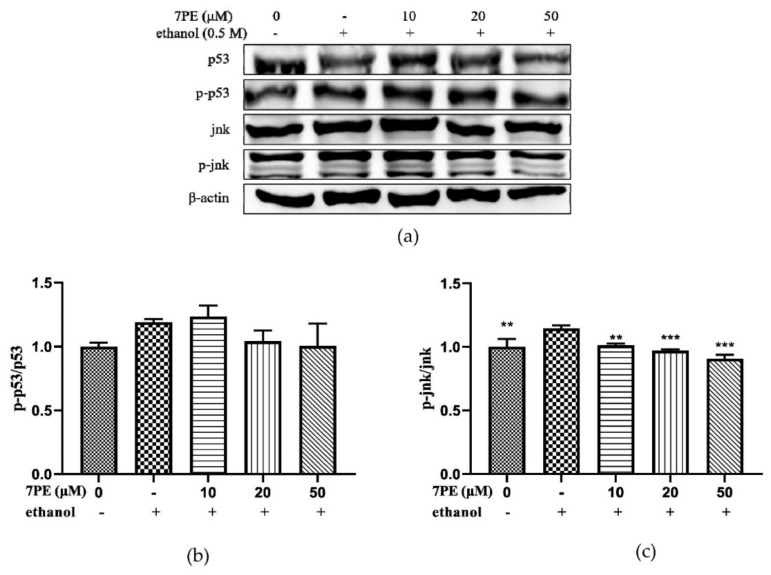
(**a**) The phosphorylation levels of p53, p-p53, JNK, and p-JNK proteins in HepG2/CYP2E1 cells. Cells were treated with 7PE (10, 20, and 50μM) for 2 h and then treated with 0.5 M ethanol for 24 h; (**b**) the ratios of p-p53/p53 were calculated; (**c**) the ratios of p-JNK/JNK were calculated. Data are shown as mean ± SD (*n* = 3). * Compared with the control group (ethanol-induced group). ** *p* < 0.01, *** *p* < 0.001.

Apoptosis depends on the activation of receptors for the mitochondrial-dependent death pathway, and the process is also affected by many other signaling pathways, such as p53 and c-Jun N-terminal kinase (JNK). Therefore, the effects of 7PE on p53 and JNK were studied (Figure 7). The results showed the phosphorylation level of JNK in the control group was significantly increased, and after 7PE treatment, the value of p-JNK/JNK showed a dose-dependent decrease. However, p53 did not change significantly. This indicated that 7PE only inhibited the phosphorylation of JNK at the protein level but had little effect on p53.

### 2.8. GSH and bcl-2 Molecular Docking Analysis

**Figure 8:**
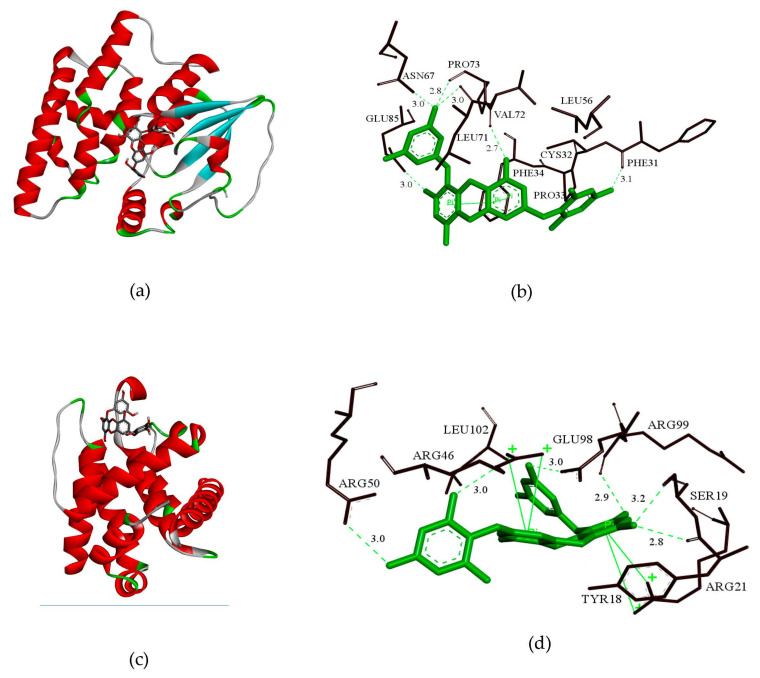
(**a**,**c**) 3D model of the interaction between 7PE and GSH and between 7PE and bcl-2, respectively. (**b**,**d**) 3D model of the optimal docking structure interaction between 7PE and the active sites of GSH and bcl-2, respectively.

In order to elucidate the structure-activity relationship of 7PE, the molecular interaction modes of GSH, bcl-2 protein, and 7PE were studied by molecular docking analysis. 7PE was docked with the active pockets of GSH and bcl-2 proteins to obtain the optimal docking structure (Figure 8a,c). The affinity of GSH and bcl-2 proteins to 7PE was −8.7 kcal/mol and −8.0 kcal/mol, respectively. As shown in Figure 8b, 7PE exhibits a tight binding pattern in the active pocket of GSH protein. 7PE was enclosed in a cavity bag composed of amino acids PHE31, CYS32, PRO33, PHE34, LEU56, ASN67, LEU71, VAL72, PRO73, and GLU85. Through a detailed analysis, it could be concluded that the four hydroxyl groups of 7PE could form six hydrogen bonds with amino acids PHE31, ASN67, LEU71, VAL72, PRO73, and GLU85, which were the main forces between 7PE and GSH. As shown in Figure 8d, 7PE was located in the active pocket composed of amino acids TYR18, SER19, ARG21, ARG46, ARG50, GLU98, ARG99, and LEU102. It is important that the five hydroxyl groups of 7PE could form six hydrogen bonds with amino acids SER19, ARG46, ARG50, GLU98, and ARG99, respectively. These hydrogen bonds were the main force between 7PE and bcl-2. All these interactions allowed 7PE to form stable complexes with GSH and bcl-2.

The aforementioned molecular docking studies provide a reasonable explanation for the interaction of 7PE with GSH and bcl-2 and lay the foundation for further research on 7PE.

## 3. Discussion

The structure of the 7PE compound was first isolated and identified by Yoshihito Okada [39] in the brown algae *Eisenia bicyclis*, and proved it had antidiabetic biological activity. However, Li [29] first isolated this structural compound in *Ecklonia cava,* and proved it had an antioxidant action. *Ecklonia cava* is an edible marine brown algae that was abundant in the subtidal areas of South Korea, Japan, and China. In addition, a variety of active substances have been extracted from the brown algae, including polysaccharides [40], carotenoids, fucoidans, and polyphenols [38]. These active substances show different biological activities in pharmaceuticals, nutraceuticals, cosmeceuticals, and functional foods, including antioxidant, anticoagulant, antibacterial, anti-human immunodeficiency virus, anti-inflammatory, and anti-tumor actions [41]. Among them, the polyphenol compounds were the main research objects of brown algae, *Ecklonia cava*.

The antioxidant activity of polyphenols is in direct relation with their chemical structures, such as the number as well as the position of the hydroxyl groups [42]. The greater the number of hydroxyl groups, the stronger the antioxidant activity of polyphenols, but as the number of hydroxyl groups increases, the stability of polyphenols will decrease [29,42]. In addition, from the perspective of molecular docking, the interaction between the polyphenols and the target protein mainly depends on the hydrogen bonding force, and the hydroxyl structure happens to be the best site for hydrogen bonding with the protein [34]. Therefore, the number of hydroxyl groups is one of the important indicators for the binding of polyphenol molecules to target proteins, and it was also one of the foundations for studying the stability and antioxidant activity of polyphenols. And among the polyphenolic compounds of *Ecklonia cava*, research on the two structures of eckol and dieckol is relatively mature, and both compounds have antioxidant activity [33,43]. According to research, eckol and dieckol have six and eleven hydroxyl groups, respectively, while 7PE has eight hydroxyl groups. Therefore, it is possible that 7PE with hydroxyl group number between eckol and dieckol will have better performance in both antioxidant activity and structural stability. This study proved that 7PE had excellent antioxidant activity and inhibited ROS-induced apoptosis.

The MTT assay (Figure 1d) showed that 7PE with a concentration of 10–100 μM had no cytotoxicity. When the concentration of ethanol stimulation is 0.5 M, the cell viability decreased by half, but when 7PE (20–100 μM) was added, the cell viability increased significantly, which showed that 7PE had an obvious relative repair effect on alcohol-induced injury. The experimental results also showed that the expression of reactive oxygen species and nitric oxide increased after ethanol treatment. However, 7PE treatment can reduce the production of reactive oxygen species (ROS) and nitric oxide (NO), increase the levels of superoxide dismutase (SOD) and glutathione protein (GSH), and reduce the level of GGT protein. Finally, the comet assay (Figure 3) showed that 7PE could reduce DNA damage caused by alcohol. Severe ALD can lead to hepatitis and liver cancer. In the process of ALD developing into a liver tumor, the growth and metastasis of liver cells need the support of a large number of cytokines and nutrients [44]. Oxidative stress can induce liver cells to secrete TNF-α, which leads to inflammation. Long-term sustained oxidative stress can lead to inflammatory responses [45] and further increase the expression of inflammatory factors. TNF-α is associated with a number of inflammatory diseases. According to ELISA results, alcohol induction will lead to the overexpression of TNF-α and inflammatory factors, but 7PE can regulate TNF-α and IL-1 and inhibit inflammation (Figure 4e,f). However, studies have shown that IL-6 is a hepatoprotective factor, which can predict alcoholic liver injury [46,47]. The experimental results also showed that the expression of IL-6 did not decrease significantly after 7PE treatment, indicating that 7PE did not decrease the content of liver-protective factors. At the same time, the amount of IL-6 did not increase, possibly due to other reasons, such as insufficient stimulation. In summary, 7PE could reduce the production of inflammatory factors, but whether it can increase the role of liver-protective factors remains to be further studied.

Studies have shown that alcohol can lead to oxidative stress and ROS overexpression [10,48], coupled with the synergistic reaction of NO. ROS and NO can act as second messengers and activate the expression of apoptotic genes, thus increasing apoptosis [43]. Apoptosis can be controlled by various apoptosis-related proteins, including bcl-2 family proteins, death receptors, and caspase [49]. Caspase-3 is considered to be the key protein in the farthest effect pathway of apoptosis [50]. In this study, Western blot detected that the cleaved caspase-3/procaspase-3 and bcl-2/bax values decreased. Many factors can activate the PI3K pathway, which leads to the activation of Akt. Akt plays an important role in cell survival signal transduction [51]. Akt can phosphorylate and inhibit the pro-apoptotic Bcl-2 family members Bad, Bax, and caspase-9. Western blot showed that the ratio of p-akt/akt increased (as observed in Figure 5d), while the ratio of cleaved caspase-9/procaspase-9 decreased (Figure 5f), which proved that 7PE could resist apoptosis by activating Akt.

Nuclear factor-κB (NF-κB), composed of proteins p50, p65, and IκB, is related to the control of apoptosis and autophagy [52]. Without being stimulated, NF-κB is located in the cytoplasm. Extracellular stimulation causes rapid phosphorylation and subsequent degradation of IκB, thus exposing the nuclear localization sequence on p50–p65 heterodimer [53]. Then, p65 protein is phosphorylated, resulting in nuclear translocation. The results of the Western blot showed that ethanol treatment increased the phosphorylation of IκB-α and p65 in HepG2 cells. 7PE inhibits apoptosis by inhibiting phosphorylation of p65 and IκB-α (Figure 6).

Although the initiation and execution of apoptosis depending on the activation of receptors for the mitochondrial-dependent death pathway, the process is also affected by many other signaling pathways, such as p53 and c-JunN-terminal kinase (JNK) from the MAPK family [54]. After DNA damage, p53 can be activated to induce Bax transcription [55], but experiments show that p53 phosphorylation has no significant change after 7PE treatment. JNK is one of the important mitogen-activated protein kinases and is involved in stress response and apoptosis [56]. The results also showed that 7PE could significantly reduce the phosphorylation of JNK in the process of apoptosis induced by alcohol (Figure 7c), which indicates that alcohol may activate MAPKs pathways, although more proof is required. Finally, the interaction of 7PE with GSH and bcl-2 was studied by molecular docking. The results showed that the hydroxyl group of 7PE could form stable hydrogen bonds with GSH and bcl-2 (Figure 8b,d). This is the main function of molecular compounds and proteins. The results provide a theoretical basis for further verifying the role of 7PE in antioxidation at the molecular level.

Combined with the experimental results of this study, it can be concluded that 7PE could repair liver injury caused by alcohol, but the specific mechanism of action should be further studied.

## 4. Materials and Methods

### 4.1. Chemicals and Materials

7-phloro-eckol (7PE, Figure 1a) was isolated from *Ecklonia cava* in a previous study [26,27,28,29]. Dulbecco’s modified Eagle’s medium (DMEM), fetal bovine serum (FBS), trypsin-EDTA (0.25%), and penicillin/streptomycin were purchased from Gibco (New York, USA). Dimethyl sulfoxide (DMSO), DCFH-DA, 4′,6-diamidino-2-phenylindole (DAPI), and 3-(4,5-Dimethylthiazol-2-yl)-2,5-diphenyltetrazolium bromide (MTT) were purchased from Sigma-Aldrich (St. Louis, MO, USA). TNF-α (EHC103a), IL-1, and IL-6 kits were purchased from Neobio Science Technology Co., Ltd. (Shenzhen, Guangdong, China). p-pi3k (17366), AKT (4691), p-AKT (4060), c-c-9 (2075s), and c9 (9508) were provided by Cell Signaling Technology (CST, MA, USA). Pi3k (SC-376112), SOD (sc-271014), GGT (sc-100746), GSH (sc-71155), p65 (sc-8008), p-p65 (sc-136548), JNK (sc-7345), p-JNK (sc-6254), GAPDH (sc-47724), β-acting (47778), and secondary antibodies (goat anti-rabbit IgG-HRP, sc-2004; goat anti-mouse IgG-HRP, sc-2005) were purchased from Santa Cruz Biotechnology Inc. (Santa Cruz, CA, USA). All other chemicals and solvents were of analytical grade.

### 4.2. Cell Culture

HepG2/CYP2E1 cells (HepG2 cells transfected with human CYP2E1 cDNA) were provided by the Cell Bank of the Chinese Academy of Sciences (Shanghai, China). HepG2/CYP2E1 cells were cultured respectively in DMEM, 10% FBS, 100 mg/mL streptomycin, and 100 U/mL penicillin in a humidified incubator of 5% CO_2_ at 37 °C.

### 4.3. Cell Viability Assay

Cells were cultured in 96-well plates (4 × 10^3^ cells/mL) for 24 h. This was changed to a fresh serum-free medium containing different concentrations of 7PE (0, 10, 20, 50, and 100 µM) for 24 h. An amount of 100 µL MTT (1 mg/mL) was added to each well, and cells were incubated for 4 h at 37 °C. Subsequently, 100 µL DMSO was added to dissolve the formazan crystals. The absorbance was measured using a microplate reader (BioTek, Winooski, VT, USA) at 570 nm.

### 4.4. Cell ROS Analysis

ROS content was directly proportional to the fluorescence intensity of DCFH-DA. Cells were cultured in 24-well plates, and 7PE (0, 1, 10, 20, 50, and 100 µM) was added for 2 h. Cells were treated with 0.5 M ethanol for 24 h in a CO_2_ incubator. Subsequently, DCFH-DA (10 µM) was added for 30 min at 37 °C in the dark. Finally, the fluorescence intensity was examined under an inverted fluorescence microscope (Olympus, Tokyo, Japan).

### 4.5. Cell NO Analysis

No content was directly proportional to the fluorescence intensity of DAF-FM-DA. Cells were cultured in 24-well plates, and 7PE (0, 1, 10, 20, 50, and 100 µM) was added for 2 h. Cells were treated with 0.5 M ethanol for 24 h in a CO_2_ incubator. Subsequently, DAF-DA (10 µM) was added for 30 min at 37 °C in the dark. Finally, the fluorescence intensity was examined under an inverted fluorescence microscope (Olympus, Tokyo, Japan).

### 4.6. Comet Assay

HepG2/CYP2E1 cells were treated with 7PE (0, 10, 20, 50, and 100 µM) and 0.5 M ethanol for 24 h in a CO_2_ incubator. Subsequently, the cells were treated with EDTA-trypsin to form a cell suspension (1 × 10^5^ cells/mL). The cell suspension (20 μL) and 1% low-melting-point agarose (LMA, 80 μL) were mixed and dropped onto 0.8% normal-melting-point agarose (NMA, 100 μL). After the gel was cured, the slides were immersed in a precooled lysate solution (2.5 M NaCl, 200 mM NaOH, 100 mM Na2EDTA, 10 mM Tris, 1%Triton X-100, and 1% sodium lauroyl sarcosinate; pH 10) at 4 °C for 90 min. The slides were then gently immersed in an alkaline electrophoresis solution (200 mM NaOH and 1 mM Na_2_EDTA; pH > 13) at 4 °C for 30 min. Next, electrophoresis (25 V; 20 min) was performed, and the slides were stained with DAPI (20 μg/mL; 20 μL) in the dark for 10 min. Finally, the fluorescence intensity was observed under an inverted fluorescence microscope (Olympus, Tokyo, Japan), and the CASP software was applied to analyzed comet images.

### 4.7. Western Blot

Total protein from the treated HepG2/CYP2E1 cells was isolated using radio immunoprecipitation assay (RIPA) lysis buffer containing 1% phenylmethylsulfonyl fluoride (PMSF) RIPA buffer. The BCA protein assay kit was used to quantify the sample. An equal amount of protein was used for electrophoresis. The target protein was transferred to a nitrocellulose (NC) membrane (Boston, MA, USA) using SDS-PAGE. The membrane was visualized by blocking for 2 h, incubating the primary antibodies, and secondary antibody incubation was performed with enhanced chemiluminescence (ECL) detection system (Syngene, Cambridge, UK).

### 4.8. Enzyme-Linked Immunosorbent Assay (ELISA)

Cells were treated with various concentrations of 7PE (0, 10, 20, and 50 µM) for 24 h. Conditional media or cell lysates were harvested in sterile tubes and centrifuged (12,000 rpm; 4 °C) for 10 min to get the supernatants. The concentration of protein was analyzed according to the manufacturer’s protocol.

### 4.9. Molecular Docking

The chemical structure of 7PE was drawn using ChemDraw (PerkinElmer, Waltham, MA, USA) (Figure 1A), then converted into a 3D structure by Chem3D (PerkinElmer, Waltham, MA, USA), and optimized using the MMFF94 force field. The 3D structure of GSH (PDB ID: 6PNN), BCL-2 (PDB ID: 4B4S) can be downloaded from the RCSB Protein Data Bank (www.rcsb.org, accessed on 25 January 2021). GSH, BCL -2, and compound 7PE were converted to a PDBQT grid using Autodock Tools (Scr ipps Research Institute, La Jolla, CA, USA). Autodock vina (Scripps Research Institute, La Jolla, CA, USA) was used for molecular docking research. In order to increase the accuracy of the calculation, the parameter exhaustiveness was set to 100, and other parameters used the default values. Finally, the constellation with the highest score was selected for analysis using PyMoL (DeLano Scientific LLC, San Carlos, CA, USA) and Discovery Studio (Biovia, Waltham, MA, USA).

### 4.10. Statistical Analysis

Image J (Version 1.46r, NIH), GraphPad Prism 5 (Graphpad Software, San Diego, CA, USA), and the CAPS (Version 1.2.3 beta1 Krzysztof Konca, CaspLab.com, accessed on 1 February 2021) were used for data analyses. All data were analyzed by one-way ANOVA accompanied by Dunnett’s multiple comparison test for group comparison. Data are expressed as the mean ± SD (*n* = 3).

## 5. Conclusions

In summary, the current research results show that 7PE could increase the expression of SOD, GSH, and IL-1, downregulate the levels of GGT, ROS, NO, TNF-α, and IL-6, and reduce DNA damage. Therefore, 7PE could alleviate the oxidative stress induced by ethanol. In addition, 7PE can prevent ethanol-induced apoptosis by upregulating the expression of bcl-2 and AKT, downregulating the expression of bax, caspase-9, and caspase-3, and inhibiting the activation of NF-κB and JNK pathways.

The results show that 7PE could protect the liver by preventing oxidative stress and apoptosis of hepatocytes induced by alcohol. Therefore, this study lays the foundation for 7PE to be used as functional liver-protecting food and preventive substance of ALD.
